# Small scale in vitro method to determine a bioequivalent equilibrium solubility range for fasted human intestinal fluid

**DOI:** 10.1016/j.ejpb.2021.08.002

**Published:** 2021-11

**Authors:** Qamar Abuhassan, Ibrahim Khadra, Kate Pyper, Gavin W. Halbert

**Affiliations:** a Strathclyde Institute of Pharmacy and Biomedical Sciences, University of Strathclyde, 161 Cathedral Street, Glasgow G4 0RE, United Kingdom; b Department of Mathematics and Statistics, University of Strathclyde, Livingstone Tower, 26 Richmond Street, Glasgow G1 1XH, United Kingdom

**Keywords:** Oral absorption, Intestinal solubility, Simulated intestinal fluid, Fasted simulated intestinal fluid, Solubility, Naproxen, Indomethacin, Phenytoin, Piroxicam, Aprepitant, Carvedilol, Zafirlukast, Tadalafil, Fenofibrate, Griseofulvin, Felodipine, Probucol

## Abstract

Drug solubility is a key parameter controlling oral absorption, but intestinal solubility is difficult to assess in vitro. Human intestinal fluid (HIF) aspirates can be applied but they are variable, difficult to obtain and expensive. Simulated intestinal fluids (SIF) are a useful surrogate but multiple recipes are available and the optimum is unknown. A recent study characterised fasted HIF aspirates using a multi-dimensional approach and determined nine bioequivalent SIF media recipes that represented over ninety percent of HIF compositional variability. In this study these recipes have been applied to determine the equilibrium solubility of twelve drugs (naproxen, indomethacin, phenytoin, piroxicam, aprepitant, carvedilol, zafirlukast, tadalafil, fenofibrate, griseofulvin, felodipine, probucol) previously investigated using a statistical design of experiment (DoE) approach. The bioequivalent solubility measurements are statistically equivalent to the previous DoE, enclose literature solubility values in both fasted HIF and SIF, and the solubility range is less than the previous DoE. These results indicate that the system is measuring the same solubility space as literature systems with the lower overall range suggesting improved equivalence to in vivo solubility, when compared to DoEs. Three drugs (phenytoin, tadalafil and griseofulvin) display a comparatively narrow solubility range, a behaviour that is consistent with previous studies and related to the drugs’ molecular structure and properties. This solubility behaviour would not be evident with single point solubility measurements. The solubility results can be analysed using a custom DoE to determine the most statistically significant factor within the media influencing solubility. This approach has a lower statistical resolution than a formal DoE and is not appropriate if determination of media factor significance for solubilisation is required. This study demonstrates that it is possible to assess the fasted intestinal equilibrium solubility envelope using a small number of bioequivalent media recipes obtained from a multi-dimensional analysis of fasted HIF. The derivation of the nine bioequivalent SIF media coupled with the lower measured solubility range indicate that the solubility results are more likely to reflect the fasted intestinal solubility envelope than previous DoE studies and highlight that intestinal solubility is a range and not a single value.

## Graphical abstract

**Figure:**
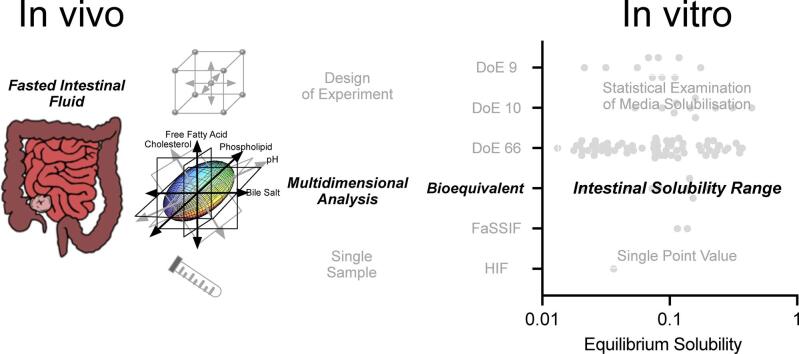

## 1 Introduction

The preferred method for the self-administration of drugs is the oral route where tablets and capsules account for over seventy percent of the marketed products available. Since solid drug is not absorbed from the gastrointestinal tract the process of drug dissolution is a critical stage during oral absorption. This is recognised in the biopharmaceutics classification system (BCS) [1] where drug solubility and permeability through the intestinal membrane are the two key parameters controlling absorption. Drugs can be categorised as exhibiting a high solubility (dose soluble in the pH range 1.2–7.5 and a fluid volume of 250 mL) or low solubility. This approach was refined in the Developability Classification System (DCS) [2], [3] through application of the dose/solubility ratio and splitting the low solubility category into two. Category IIa, for drugs where a dissolution rate limitation was likely and IIb where a solubility limited absorption would be the dominant feature. Knowledge of a drug’s solubility and position within the BCS/DCS, especially for poorly soluble drugs [4], is therefore an important parameter during drug development and for the application of quality by design concepts to the formulation and development of oral products [2].

An obvious route to the determination of a drug’s solubility in human intestinal fluid (HIF), is to use sampled intestinal fluid [5]. Fasted HIF sampling requires normal volunteers to fast overnight, which is followed by the insertion of an oral catheter and then determination of its anatomical position to ensure that it is in the small intestine. Intestinal fluid is then collected by application of a vacuum for a period of time, typically 1–2 h [6]. The volume of fluid collected depends on the protocol and volunteer and post collection has to be frozen to preserve properties. Several groups have applied this procedure [7], [8] to determine a range of drugs’ solubility in fasted [9] HIF samples. These studies have demonstrated the issues around the routine collection of HIF, the variability of the measured solubility due to variations in the HIF composition [10], [11], [12] and indicate that this approach will not provide a reproducible solubility determination. In addition the HIF sampling process limits the sample volumes available.

To circumvent HIF availability issues simulated intestinal fluids (SIF) have been developed [13] based on the components of HIF and with the fluid designed to match HIF solubility values [7], [8]. Multiple recipes are available in the literature [14] displaying variations in the content of bile salt, phospholipid, pH, buffer salt and presence or absence of additional components. This provides variability in the solubility value determined [15] by the different SIF media and presents an additional question of which media recipe is appropriate. As part of the EU IMI Oral Biopharmaceutical Tools research program [16] this group conducted a design of experiment (DoE) study into equilibrium solubility in simulated fasted media [17]. This examined seven media components (bile salt, lecithin, buffer, salt, pH, enzyme and fatty acid) using a fractional factorial design that required 66 experiments. A similar study was also conducted using six components (bile salt, lecithin, pH, buffer capacity, osmolarity and bile salt phospholipid ratio) and a different DoE protocol that required 24 experiments [18]. These studies [17], [18], [19], [20], [21], [22] quantified the significance of individual media factors for drug solubilisation, and permitted a simple classification of drug behaviour based on acidic, basic or neutral properties. For acidic drugs pH was the major driver of solubility by around a factor of 10 when compared to other media components and for basic and neutral drugs an almost equivalent combination of pH, bile salt, phospholipid and fatty acid controlled solubility. The studies also identified that there were two-way interactions between the media components that influence solubility and a further study also hinted at three-way component interactions [20], [23] affecting solubility. Indicating that these simulated systems and therefore also the natural systems exhibit complex drug solubilisation behaviour, that is difficult to fully re-capitulate using small numbers of components. Although wonderful at revealing and quantifying drug solubilisation, the DoE approaches are experimentally cumbersome [17], [18], [23] and not likely to be applied on a routine basis. To reduce experimental load, low experimental number DoE studies have been performed utilising a mixed dual level (fasted and fed) [19] or a full range (fasted + fed) designs [21] that only required 20 or 32 experiments respectively. A further dual level modification has been published that only requires 9 experiments for the fasted state [22]. Whilst these protocol adaptations reduce experimental load they remain based on a DoE, which aims to statistically determine solubility variation using conditions that are hypothesized to reflect the component variation within the experimental system or simulated fluid. Statistically this links a high concentration value of one component with a low concentration value of another (see Fig. 4 in [17]) a combination in the SIF system (for example bile salt with phospholipid) that may not arise in vivo in HIF and therefore be bioequivalent. DoE approaches therefore do not have a direct relationship to HIF.

In order to address the issues with SIF and DoE approaches a recent publication has examined HIF composition using a multidimensional mathematical analysis that treated the fluid as a 5 dimensional system [24] consisting of the following components, pH, bile salt, phospholipid, fatty acid and cholesterol. This utilised a data set of fasted and fed HIF samples obtained from volunteers [25] and identified 8 bioequivalent media compositions that statistically characterised over 90% of the variation within the sample set in the fasted and fed states. In addition a centre point was identified in each state using a Euclidean approach in 5-dimensional space, rather than the mean (or similar) value for each component since the component distributions were not normal. To achieve the multidimensional analysis the measured concentrations of components were summed and treated as a single variable, for example six bile salt species were analysed but only a single concentration calculated (Table 1). This simplification applies to bile salts, phospholipids and fatty acids were in HIF multiple species will be present. This is a situation also applicable to SIF and for bile salts it is known that the concentration has a greater influence on solubilisation than species [26]. However, it does represent an ever present challenge between simulation by simplification and the native fliud.

**Table 1:** Bioequivalent media compositions.

Media	Bile Salt (mM)	Phospholipid (mM)	FFA (mM)	Cholesterol (mM)	pH
1	1.06	0.16	1.04	0.01	6.64
2	11.45	2.48	2.88	0.38	7.12
3	3.4	0.33	2.88	0.09	8.04
4	3.56	1.18	1.04	0.06	5.72
5	3.62	1.25	3.43	0.03	7.14
6	3.35	0.31	0.87	0.17	6.62
7	5.33	0.4	2.96	0.07	6.42
8	2.27	0.96	1.01	0.08	7.34
centre point (9)	3.46	0.52	1.64	0.032	6.54

Values from [24].

In this paper we have applied the calculated fasted state compositions from the multidimensional analysis to determine the equilibrium solubility of the twelve drugs (naproxen, indomethacin, phenytoin, piroxicam, aprepitant, carvedilol, zafirlukast, tadalafil, fenofibrate, griseofulvin, felodipine, probucol) investigated in the original DoE study [17]. The equilibrium solubility data has also been compared, to the original DoE [17] and where appropriate, to the reduced experiment fasted DoE distributions [19], [22] and literature HIF and SIF values. The aim of this study is to provide a comparison of these two approaches into investigating the solubility of drugs in simulated fasted intestinal fluid systems.

## 2 Materials and methods

### 2.1 Materials

Sodium taurocholate, cholesterol, sodium chloride (NaCl), sodium oleate, ammonium formate, potassium hydroxide (KOH), hydrochloric acid (HCl), naproxen, phenytoin, piroxicam, fenofibrate, probucol, griseofulvin, carvedilol, tadalafil, and indomethacin were purchased from Merck Chemicals Ltd. Aprepitant and felodipine were provided through OrBiTo by Dr. R. Holm, Head of Preformulation, Lundbeck, Denmark. Zafirlukast was purchased from Stratech Scientific Ltd. Phosphatidylcholine from soybean (lecithin) was purchased from Lipoid company. Chloroform from Rathburn Chemical Company. FaSSIF-v1 media was purchased from Biorelevant.com Ltd. Sodium phosphate monobasic monohydrate (NaH2PO4·H2O) and formic acid from Fisher Scientific. All acetonitrile (ACN) and methanol (MeOH) solvents were HPLC gradient (VWR). All water was ultrapure Milli-Q.

### 2.2 Methods

#### 2.2.1 Solubility media preparation

Biorelevant media stock solutions

For each media recipe (Table 1) a concentrated lipid stock was prepared. The required (×15) weight of bile salt (sodium taurocholate), phospholipid (soybean lecithin) and fatty acid (sodium oleate) for each media recipe was dissolved in chloroform (3 mL) – stock A. The required weight of cholesterol (×1500) for each media recipe was dissolved in chloroform (10 mL) – stock B. An aliquot of stock B (0.1 mL) was added to each stock A, mixed and the stock A chloroform solution evaporated under a stream of dry nitrogen gas. The dry lipid film was resuspended in water, quantitatively transferred to a volumetric flask (5 mL) and made to volume with water. Stock aqueous solutions of buffer (sodium phosphate monobasic monohydrate; 28.4 mM) and salt (sodium chloride; 105.9 mM) were prepared in water.

Fasted simulated small intestine fluid (FaSSIFv1) media

Pre-prepared media from Biorelevant company was used as described by the manufacturer.

#### 2.2.2 Equilibrium solubility measurement

The method was based on multiple previous papers [17], [21], [22]. Into a centrifuge tube (15 mL Corning® tubes) was added aliquots (267 µL) of the lipid, buffer and salt stock solutions, an excess of the solid drug under test and water (3.199 mL) to make a final aqueous system volume of 4 mL. Tube pH was adjusted to the required value (Table 1, target value ± 0.05) using KOH or HCl as required. FaSSIF-v1 media (4 mL) was added to the tube along with an excess of the solid drug under test and pH adjusted if required. The tubes were capped and placed at room temperature into an orbital shaker (Labinco BV model L28) for 1 h, and the final pH was re-adjusted if required. Tubes were then placed in the shaker at 37 °C for 24 h. Post incubation an aliquot (1 mL) of each tube was transferred to a 1.5 mL Eppendorf tube, and centrifuged for 15 min, 10000 rpm and the supernatant analysed by HPLC for drug content. For each drug this process was repeated three times and the average value is used.

#### 2.2.3 HPLC analysis

Analysis was performed on a Shimadzu Prominence-i LC-2030C HPLC system using a gradient method for all the drugs except probucol. Column Xbridge® C18 5 µm (2.1 × 50 mm) at 30 °C, mobile phase A 10 mM ammonium formate pH 3 (adjusted with formic acid) in water, and mobile phase B 10 mM ammonium formate in acetonitrile:water (9:1), flow rate 1 mL/min (except carvedilol 0.7 mL/min), gradient start 70:30 (A:B), 3 min 0:100, 4 min 0:100, 4.5 min 70:30 total run time 8 min. The retention time, analysis wavelength and injection volume for each drug are provided in Table 2. For probucol an isocratic method was used [17] mobile phase ACN, MeOH, and water 45:45:10 and the column was Speck and Burke, ODS-H optimal 5 µm (30 × 150 mm). For each drug a concentration curve was prepared using five or six standards that bracketed all the measurement concentrations, for all drugs correlation coefficient > 0.99.

**Table 2:** HPLC conditions.

Drug	Retention time (min)	Wave-length (nm)	Injection volume (µL)
Naproxen	1.6	254	10
Indomethacin	2.1	254	10
Phenytoin	1.1	254	20
Piroxicam	1.07	254	10
Aprepitant	2.27	254	50
Carvedilol	1.6	254	10
Zafirlukast	2.6	254	25
Tadalafil	1.4	291	50
Fenofibrate	3	291	10
Felodipine	2.4	254	10
Griseofulvin	1.5	291	10
Probucol	4.87	220	100

#### 2.2.4 Data analysis

Data comparison using non-parametric Kruskal-Wallis test with Dunn’s multiple comparison correction was conducted in Prism 9 for MacOSX, only comparisons indicated in the figures was analysed. Media bioequivalent factor concentrations/values (Table 1) was used as an input for a factorial custom design of experiment using Minitab®19 and the significant factors influencing solubility calculated.

## 3 Results and discussion

### 3.1 Equilibrium solubility

**Fig. 1:**
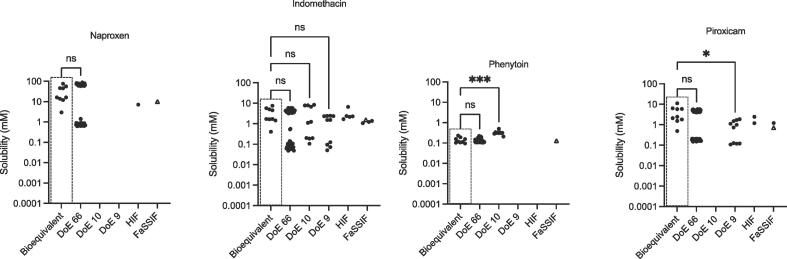
Measured Equilibrium Solubility of Acidic Drugs. Bioequivalent – this study; DoE 66 [17]; DoE 10 [19]; DoE 9 [22]; HIF (Fasted Human Intestinal Fluid) data from [10]; FaSSIF (Fasted Simulated Intestinal Fluid) data from [10], plus one point (Δ) from this study. ns = no significant difference; * p = 0.0172; *** p = 0.0003.

**Fig. 2:**
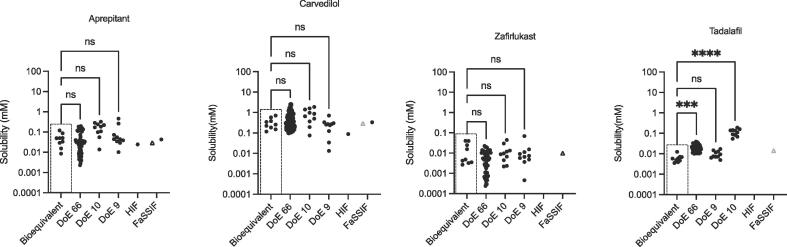
Measured Equilibrium Solubility of Basic Drugs. Bioequivalent – this study; DoE 66 [17]; DoE 10 [19]; DoE 9 [22]; HIF (Fasted Human Intestinal Fluid) data from [10]; FaSSIF (Fasted Simulated Intestinal Fluid) data from [10], plus one point (Δ) from this study. ns = no significant difference; *** p = 0.0002; **** p = 0.0001.

**Fig. 3:**
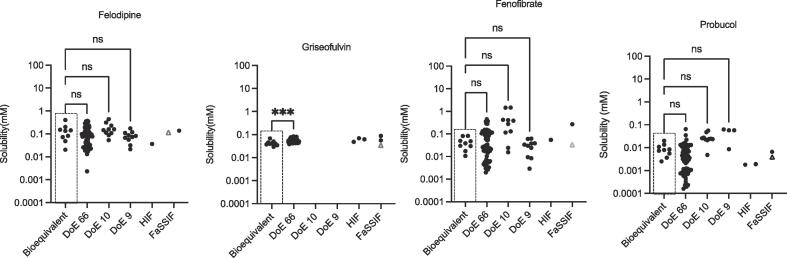
Measured Equilibrium Solubility of Neutral Drugs. Bioequivalent – this study; DoE 66 [17]; DoE 10 [19]; DoE 9 [22]; HIF (Fasted Human Intestinal Fluid) data from [10]; FaSSIF (Fasted Simulated Intestinal Fluid) data from [10], plus one point (Δ) from this study. ns = no significant difference; *** p = 0.0006.

The equilibrium solubility results from this bioequivalent nine point fasted study are presented in Fig. 1 for the acidic drugs and in Fig. 2, Fig. 3 for the basic and neutral drugs. For each drug the comparable data set from the initial 66 point DoE (DoE 66) fasted study [17] is included along with, where available, results from the smaller sample number fasted DoE studies, DoE 10 [19] and DoE 9 [22]. Literature values for equilibrium solubility in fasted HIF or fasted SIF media [10] (NB One FaSSIF value is from this study) are provided for visual comparison but are not included in the statistical analysis.

A statistical comparison of the bioequivalent equilibrium solubility distribution with the DoE results (Fig. 1, Fig. 2, Fig. 3) indicates that in the thirty cases where a comparison is possible, twenty five (just over 80%) are statistically equivalent. Indicating that in the majority of cases the bioequivalent approach is measuring the same solubility space as the previous DoE approaches. A comparison against available HIF solubility values indicates that of the nine possible drug-based comparisons the literature fasted HIF equilibrium solubility data points lie within the bioequivalent envelope in seven cases, almost eighty percent. A similar analysis of the fasted SIF equilibrium solubility values indicates that in nine out of twelve (seventy five percent) possible drug based comparisons, the data points lie within the bioequivalent solubility envelope. The comparisons against literature HIF and SIF values contain an unavoidable error since multiple protocols have been applied in the determination of these values. However, the HIF and SIF comparison provides a similar level of agreement (approximately eighty percent) with the DoE comparison and collectively indicates that in the majority of cases the bioequivalent approach is measuring the same equilibrium solubility space as literature DoE, HIF and SIF approaches.

**Fig. 4:**
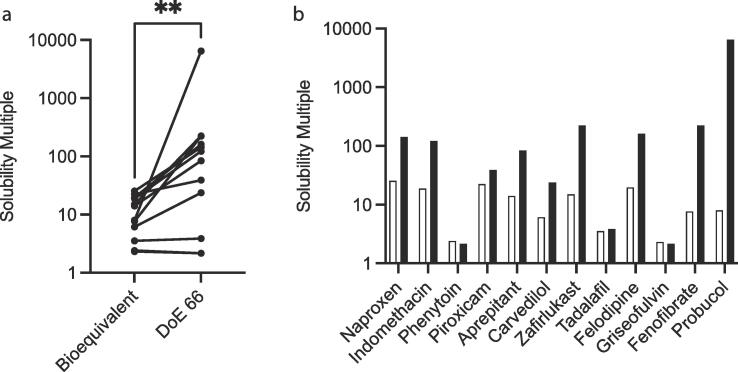
a. Collected Solubility Multiple Values. Bioequivalent – this study; DoE 66 [17]; ** p = 0.0024. Solubility multiple (highest measured solubility ÷ lowest measured solubility). b. Individual Solubility Multiple Values. Bioequivalent – this study – open bar; DoE 66 [17] – closed bar. Solubility multiple (highest measured solubility ÷ lowest measured solubility).

A striking feature of the original DoE 66 was equilibrium solubility variability, with some drugs exhibiting a greater than three log range between the lowest and highest values measured. In Fig. 4a the calculated solubility multiple (highest solubility ÷ lowest solubility) is presented for each drug in the DoE 66 and bioequivalent test systems. There is a statistically significant reduction in the solubility multiple in the bioequivalent system where for nine out of the twelve drugs the value is smaller. There is no available comparison with literature data but there are several possible reasons for this result. The bioequivalent system only contains nine measurement points and therefore the possibility for variability is lower, but will depend upon the variability of the media compositions examined. The range of media factors and factor values assessed between the systems is not equivalent and this will influence the solubility measurements, for example the DoE 66 pH range was between 5 and 7, whilst the bioequivalent range is greater at between 5.7 and 8. In contrast the fatty acid range is lower in the bioequivalent (0.9–3.4 mM) when compared to the DoE 66 (0.5–10 mM). In addition cholesterol is present in the bioequivalent system but not in the DoE 66. The combined solubility influence of these various differences is difficult to predict. However, the pH difference between DoE systems (all pH range 5–7) with the bioequivalent system (pH range 5.7–8) is probably the reason for the statistical difference determined for piroxicam in DoE9 (Fig. 1). No difference is detected for piroxicam in the bioequivalent with the DoE 66 due to the difference in the data point numbers. Finally, the bioequivalent system does not contain statistically driven measurement points that combine a high value of one factor with a low value of another (see Introduction). It is known from the previous high number DoE systems that media factors interact [17], [20] to influence solubility. This is likely to produce increased solubility variability but would require a more detailed analysis to separate this effect out from points that do not contain this issue. Overall the bioequivalent system is providing a reduced solubility range that due to the method applied to derive the measurement points’ composition [24] represents a more realistic fasted intestinal solubility window than DoE based investigations into the media, its factors and factor ranges.

There are three drugs (phenytoin, tadalafil and griseofulvin) where the difference in the solubility multiple between the two systems is minimal (Fig. 4b). These drugs also have the lowest solubility multiple values and represent three of the four drugs (and four of the five cases) where there is a statistical difference between the solubility data sets. This multi-point assessment process reveals a behaviour that has not been previously reported in the literature, possibly since studies only examine a single point or SIF recipe [27] but with multiple drugs. The behaviour is different to the rest of the drug test set and surprisingly is one example from each of the three drug categories (acidic, basic and neutral) examined. The solubility distributions indicate that these drugs have a very low solubility variability within a simulated intestinal media system and presumably therefore HIF, and the solubility window moves as the media factors and factor values are varied. The latter statement is self-evident but the consistent low solubility range is not and overall this result is an example of drug dependent solubility behaviour in these systems, which is present [17], [20], but is very difficult to visualise [20], [23]. It is interesting that these three drugs have relatively a low molecular weight and log P value and molecularly have similar compact structures with predominantly flat aromatic rings. This simple chemical property analysis could also be applied to naproxen (pKa 4.15), indomethacin (pKa 4.5) and piroxicam (pKa 6.3), but the solubility multiple for these drugs is much larger (Fig. 4b). However, for the acidic drugs it is known that pH is the major solubility driver [17] and these drugs have pKa values within the DoE 66 or bioequivalent pH range. This is evident in the DoE 66 results (Fig. 1) where points cluster in either high or low groups (pH values tested 5, 6 and 7), however the solubility multiple within a pH cluster is low. It is interesting that this low solubility multiple is present in both ionised and non-ionised states for naproxen, indomethacin and piroxicam. The limited solubility variability in the ionised state is understandable, since this represents aqueous solubility of the ionised molecule, but the tight solubility of the non-ionised which should partition into the amphiphilic micellar structure is comparable to the behaviour of phenytoin (pKa 8.33), tadalafil (0.85) and griseofulvin (neutral), with the latter two not ionised. This is most likely to be related to molecular structure and properties and indicates that molecular structure sits within the three categories in controlling solubility behaviour in fasted intestinal media systems. There are not sufficient examples in this study to assess this effect, however this is an indication of a link between molecular structure or shape and solubility in the intestinal media systems over and above more general properties such as pKa and log P [28]. Further more focussed studies will be required to fully elucidate this behaviour.

### 3.2 Media factor analysis

Although the bioequivalent media composition is based on a multi-dimensional analysis of fasted intestinal media [24] it is possible to fit the factor values into a tailored DoE structure [21]. This allows a standardised effect value to be calculated for the impact of each media factor on drug solubility, but does not permit the calculation of two-way or higher effects. The results are presented in Table 3 along with effect values from the three previous equilibrium fasted solubility DoE studies [17], [19], [22]. For the bioequivalent system significant media factors were detected for eight out of the twelve drugs (sixty seven percent). This rate is lower than either of the two reduced number DoEs (note comparison only based on the drugs analysed in this study and present in either DoE) which are at seventy seven percent (DoE 10 [19]) and eighty seven percent (DoE 9 [22]), whilst the large number DoE (DoE 66 [17]) is at one hundred percent. The lower number of factors identified when comparing DoE 66 to DoE 10 or DoE 9 can be attributed to the lower number of experimental points in these systems reducing the statistical power of the experimental design [22]. The further reduction in the bioequivalent system can be attributed to the fact that the experimental points measured are also not statistically designed for the DoE process.

**Table 3:** Significant media factors affecting compound solubility in the systems.

	Bioequivalent	DoE 66	DoE 10	DoE 9
Naproxen	pH	pH	NT	NT
Indomethacin	pH	pH, bile salt, buffer, oleate	pH	pH
Phenytoin	NSF	pH, bile salt, lecithin, oleate, buffer, salt, pancreatin	pH, oleate, cholesterol, BS:PL ratio	NT
Piroxicam	pH	pH	NT	NT
Aprepitant	lecithin, oleate	oleate, pH, lecithin	Oleate, lecithin, monoglyceride	NSF
Carvedilol	lecithin	bile salt, oleate	NSF	bile salt, pH
Zafirlukast	NSF	pH, oleate, lecithin, bile salt	pH, cholesterol, monoglyceride	pH, oleate, bile salt, lecithin
Tadalafil	lecithin	bile salt, pH, buffer, lecithin, oleate, salt	NS	pH
Fenofibrate	lecithin	oleate, bile salt, pH, lecithin, buffer, salt	pH, oleate, lecithin	oleate
Felodipine	lecithin	pH, oleate, lecithin, bile salt	pH, oleate, lecithin, monoglyceride	oleate
Griseofulvin	NSF	pH, bile salt, lecithin, oleate, buffer, salt	NT	NT
Probucol	NSF	pH, oleate	Oleate, BS:PL ratio	pH

NT: drug not tested in this system.

NSF: no significant factors detected.

The majority of factors identified (eight out of nine) in the bioequivalent analysis are identified by DoE 66 with the one exception for carvedilol where lecithin is the sole significant biorelevant factor but is not identified in DoE 66. A comparison with the small scale DoEs indicates that the correlation is reduced to below 50% and in some cases detection of factor significance is variable and there are several reasons for the differences. The already reduced statistical power of the smaller number of experiments and the variations in the factors present within each media system (see comment on cholesterol above) combined with variations in the levels of the factors (see comments on pH and oleate above).

Since the bioequivalent system was designed not to be a DoE the reduced number of factors identified and limited number of correlations with DoE results is to be expected. The identification of a significant factor that corresponds to the DoE 66 factors could therefore be considered a bonus and if identification of media factors influencing solubility is required a DoE approach is preferable.

## 4 Conclusions

This study demonstrates that it is possible to assess the fasted intestinal equilibrium solubility distribution using a small number of bioequivalent media recipes obtained from a multi-dimensional analysis of sampled fasted human intestinal fluid. The solubility distribution obtained is statistically equivalent to those determined using DoE studies, which indicates that this approach is examining the same solubility space. In addition, the data from this paper in combination with the results from multiple design of experiment papers [17], [18], [19], [22] and other single point solubility measurements [10] indicates that the use of simulated media system, utilising the same media factors and concentrations are likely to provide similar solubility distributions.

By creating a custom design of experiment using the bioequivalent media recipe factor values it is possible to calculate the factors significantly influencing drug solubility. However, the number of factors identified is reduced when compared to statistically designed small scale studies [19], [22], which are again lower than the large scale studies [17]. Therefore small scale studies using bioequivalent media compositions are not useful for the identification of the media factors or factor combinations that significantly influence a drug’s solubility and to assess this property large scale DoE studies are required.

For three drugs (phenytoin, tadalafil and griseofulvin) this study identifies a very narrow solubility distribution that is consistent with behaviour in previous studies [17], [19], [22]. This indicates that molecular structure impacts solubilisation in these systems on top of basic physicochemical parameters such as pKa and log P. However, there is insufficient data within this study to fully analyse this result. The detection of this behaviour was only possible through the application of multiple point solubility studies rather than the single point studies more commonly applied [28]. This might indicate that in order to understand and predict intestinal solubilisation behaviour of drugs, multiple point solubility assessments should be applied.

The solubility variability measured by this study is statistically significantly lower than the variability from the initial large scale design of experiment [17] study. The two studies are not directly comparable and multiple factors could be responsible for this difference. However, based on the source for the media recipe compositions in this study, the lower solubility range measured is more likely to reflect the fasted intestinal solubility envelope than a design of experiment approach. The results also indicate that intestinal solubility is a range, not a single point, and this should be accounted for when assessing solubility impact in the BCS or DCS. Further studies would be useful in an attempt to link this in vitro measurement with in vivo performance and also other important biopharmaceutical properties such as dissolution and supersaturation.

## Declaration of Competing Interest

The authors declare that they have no known competing financial interests or personal relationships that could have appeared to influence the work reported in this paper.
